# Applications and challenges of biomaterial mediated mRNA delivery

**DOI:** 10.37349/etat.2022.00093

**Published:** 2022-08-31

**Authors:** Huapan Fang, Qian Chen

**Affiliations:** Institute of Functional Nano & Soft Materials (FUNSOM), Jiangsu Key Laboratory for Carbon-Based Functional Materials & Devices, Soochow University, Suzhou 215123, Jiangsu, China; Donghua University, China

**Keywords:** Messenger RNA therapeutics, delivery efficiency, messenger RNA vectors, routes of administration, storage methods, safety

## Abstract

With the rapid development of gene therapy technology and the outbreak of coronavirus disease 2019 (COVID-19), messenger RNA (mRNA) therapeutics have attracted more and more attention, and the COVID-19 mRNA vaccine has been approved by the Food and Drug Administration (FDA) for emergency authorization. To improve the delivery efficiency of mRNA *in vitro* and *in vivo*, researchers have developed a variety of mRNA carriers and explored different administration routes. This review will systematically introduce the types of mRNA vectors, routes of administration, storage methods, safety of mRNA therapeutics, and the type of diseases that mRNA drugs are applied for. Finally, some suggestions are supplied on the development direction of mRNA therapeutic agents in the future.

## Introduction

Gene therapy, as a novel therapeutic method, is used to treat cancer, genetic diseases, infectious diseases, and other diseases [1–3]. Among them, messenger RNA (mRNA)-based therapeutics as vaccines against coronavirus disease 2019 (COVID-19) have been urgently authorized by the Food and Drug Administration (FDA) in the United States. mRNA was discovered in the 1960s and the *in vitro* mRNA transcription entered a rapid development in the late 1980s [4, 5]. Moreover, *in vivo* transfection of mRNA has been studied since the 1990s [6]. In general, the naked mRNA is negatively charged and belonged to macromolecules, which could not be effectively taken up by target cells because of the negatively charged cell membranes [7, 8]. Moreover, even though the mRNA cargo is taken up by the target cells and enters the endosomes, the mRNA needs to escape from the endo/lysosomes and enter the cytoplasm for gene transfer. Therefore, efficient vectors are essential for successful mRNA delivery [9–18].

With a variety of mRNA delivery systems undergoing development, mRNA-based therapeutics have showed promising application prospects including viral vaccines, cancer therapy, cardiovascular diseases, and other types of diseases [19–24]. To improve the delivery efficiency of mRNA-based therapeutics *in vitro* and *in vivo*, various mRNA carriers are developed, including lipid nanoparticles (LNPs) [25], cationic peptides [26–28], polymers [8, 29–32], and other types of biomaterials [33–36]. It is expected that mRNA can specifically reach target cells and efficiently express functional proteins. It is worth mentioning that mRNA vaccines, including mRNA-1273 and BNT162b2, had been urgently authorized by FDA as COVID-19 vaccines, further promoting the great development of mRNA drugs [37–39]. At present, there are many other mRNA-based formulations in clinical trials trial stage [22].

**Figure 1.**
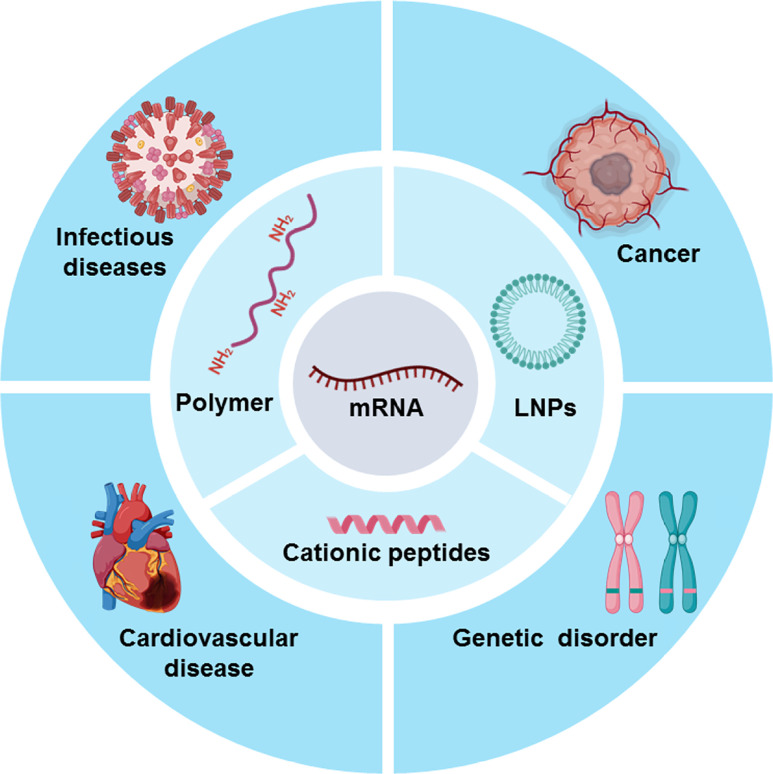
mRNA carriers and disease types of mRNA drug action

To improve the delivery efficiency of mRNA drugs, a variety of mRNA carriers have been designed and developed, including LNPs, cationic peptides, polymers, and other types of carriers. Moreover, to maximize the efficacy of mRNA therapeutics, the administration routes of mRNA therapeutics are explored according to the types of diseases. Additionally, the mRNA stability, storage conditions, and *in vivo* safety of mRNA therapeutic agents are also urgently needed to be considered. In this review, we will briefly introduce the mRNA carriers for mRNA delivery (Figure 1); the key steps of preclinical development of mRNA-based therapeutics include administration routes, storage methods, and safety, and mRNA drugs for disease treatment. Finally, we put forward some suggestions on the development of mRNA therapeutics and the directions of mRNA drugs in the future.

## mRNA carriers

### LNPs

**Figure 2.**
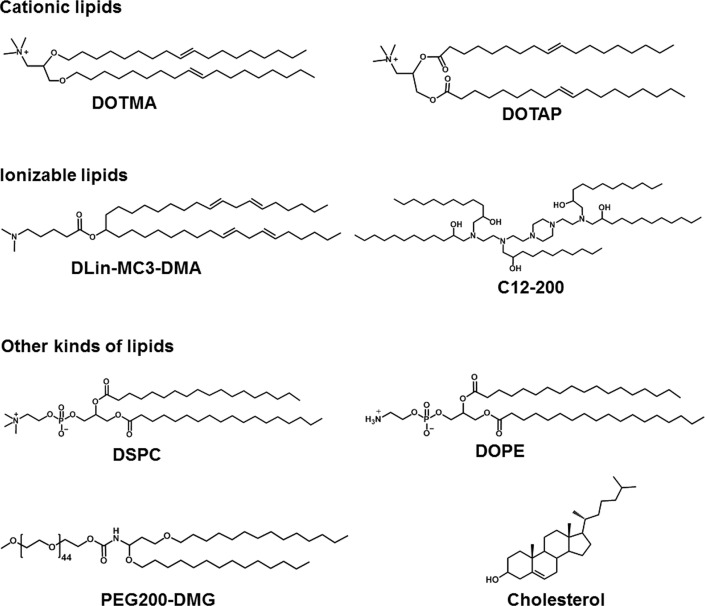
Representative cationic lipids, ionizable lipids, and other kinds of lipids in mRNA formulations. DLin-MC3-DMA: dilinoleylmethyl-4-dimethylaminobutyrate; DMG: dimyristoylglycerol; DSPC: 1,2-distearoyl-*sn*-glycero-3-phosphocholine; PEG: polyethylene glycol

LNPs are served as smart nano-sized carriers for mRNA delivery *in vitro* and *in vivo*. In general, lipids are amphiphilic molecules and contain three parts: a polar head group, a hydrophobic tail, and a linker between the two parts. However, LNPs for mRNA delivery usually contain cationic lipids, ionizable lipids, or other types of lipids (Figure 2) [25].

The head group of cationic lipids is permanently positively charged [23] and can condense the negatively charged mRNA into nanoparticles. For example, Malone et al. [5] developed cationic lipid *N*-[1-(2,3-dioleyloxy)propyl]-*N*,*N*,*N*-trimethylammonium chloride (DOTMA) for mRNA delivery. In human, rat, mouse, and *Drosophila* cells, carrier/mRNA showed efficient gene transfection. Additionally, 1,2-dioleoyl-3-trimethylammonium-propane (DOTAP), an analogue of DOTMA, has been applied either along or together with other materials for mRNA delivery. Kranz et al. [40] used cationic lipid DOTMA and 1,2-dioleoylphosphatidylethanolamine (DOPE) to construct a cationic liposome (Lipo) to load mRNA encoding tumor antigen. The resulting Lipo/mRNA (RNA-Lipo) could efficiently mediate mRNA uptake and induce the antigen expression by dendritic cells (DCs) and macrophages. RNA-Lipo encoding tumor antigens induced strong effector and memory T cell responses, mediating an excellent inhibition of progressive tumors. Wang et al. [41] designed a cationic Lipo composted of DOTAP and cholesterol at a molar ratio of 1:1, and such Lipo could achieve effective encapsulation of protamine/mRNA through electrostatic interaction with carrier/mRNA complexes. Modified mRNA encapsulated by Lipo-protamine-RNA (LPR) showed significantly increased cellular uptake by tumor cells, and LPR exhibited significant improvement compared with the equivalent plasmid DNA (*p*DNA). In the H460 xenograft-tumor model, mRNA encoding herpes simplex virus 1-thymidine kinase served as a therapeutic gene, systemic administration of LPR considerably inhibited the tumor growth. Moreover, Lei et al. [42] used mRNA encoding interleukin 15 (*IL-15*) as a therapeutic gene; in the orthotopic CT26 colon cancer model, intraperitoneal and intravenous administration of carrier/mRNA encoding *IL-15* could effectively inhibit tumor growth and lung metastasis by inducing systemic antitumor immune response.

For ionizable lipids, each ionizable lipid had its own acid dissociation constant (pK_a_). Once the pH value was less than pK_a_, these ionizable lipids were protonated and positively charged; when the pH value was higher than pK_a_, these ionizable lipids were unionized [43]. Due to toxicity issues of cationic lipids, ionizable lipids served as alternatives. In general, we hoped that ionizable lipids were non-ionized under physiological conditions (pH 7.4), so as to reduce their systemic toxicity in the blood circulation; on the other hand, the ionizable lipids should be protonated in early endosomes (pH 5.5–6.0) and could promote the endo/lysosomal escape of lipid/mRNA complexes into cytoplasm for mRNA translation [44]. Recently, FDA approved the first RNA drug (patisiran) for the treatment of hereditary transthyretin protein amyloidosis (hATTR), which had an ionizable lipid called DLin-MC3-DMA [45]. Additionally, other scientists also used DLin-MC3-DMA to mediate mRNA for expressing therapeutic proteins including human erythropoietin and frataxin *in vivo* [46, 47]. Finn et al. [48] used an ionizable lipid as a part of LNPs to co-deliver Cas9 mRNA encoding transthyretin and single guide RNA (sgRNA), successfully editing mouse transthyretin gene in the liver. Ramaswamy et al. [49] developed lipid-enabled and unlocked nucleic acid modified RNA as a safe and effective LNP mRNA delivery platform. Among them, an ionizable lipid, Arcturus Therapeutics’ lipid (ATX) was one component of LNPs for mediating mRNA encoding human factor IX protein to treat hemophilia [49]. Moreover, An’s group [50] and Sabnis’ group [51] used biodegradable ionizable lipids for mRNA delivery *in vivo*. Kauffman et al. [52] designed diketopiperazine-based ionizable lipid C12-200 for mRNA delivery. Moreover, Oberli’s group [53] and Yin’s group [54] used C12-200 lipid to mediate mRNA expressing therapeutic protein for cancer immunotherapy and genome editing, respectively.

Except for cationic or ionizable lipids, the LNPs typically also included other lipid components, such as phospholipids, PEG-functionalized lipids (PEG-lipids), or cholesterol. These lipids were used to improve particle stability, tolerability, and biodistribution [20, 44, 55]. DSPC as a phospholipid, had saturated tails, and the cylindrical geometry contributed DSPC molecules to form a lamellar phase for stabilizing LNPs [56, 57]. DSPC was included in mRNA-1273 and BNT162b2, the two COVID-19 vaccines. DOPE contained two unsaturated tails, and its conical shape tended to an inverted hexagonal H(II) phase, which destabilized endo/lysosomal membranes and facilitated endosomal escape of LNPs. Moreover, PEG-lipids also affected the delivery properties of LNPs [57–60]. PEG-lipids could relieve particle aggregation and stabilize LNPs. In addition, PEG segment prolonged the blood circulation time by means of reducing the clearance of kidneys and mononuclear phagocyte system [58–60]. The extent of these effects depended on the proportions and properties of the PEG-lipids (such as lipid length and chain length of PEG) [20, 57]. Cholesterol could enhance particle stability by modulating membrane integrity and rigidity [55]. The geometry of cholesterol derivatives also affected the delivery efficacy and biodistribution of LNPs. For example, LNPs contained cholesterol derivatives adopting a polyhedral shape, which had multilamellarity and lipid partitioning [61]. Moreover, oxidation of cholesterol tail enabled more LNPs to accumulate in liver endothelial cells and Kupffer cells instead of hepatocytes [62].

### Cationic peptides

**Figure 3.**
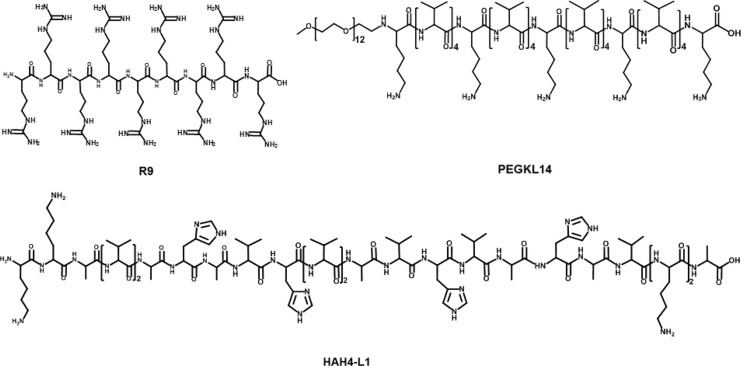
Representative cationic peptides for mRNA delivery

Cationic peptides have been used as carriers to load nucleic acid for its intracellular delivery (Figure 3) [63]. Among them, cationic cell-penetrating peptides (CPPs)-based mRNA delivery systems were developed for promoting T cell immunity response *in vivo*. For example, Feiner-Gracia et al. [64] used R9 peptide to load mRNA to form CPP-mRNA polyplexes and unveiled the impact of peptide stoichiometry on the destabilization of mRNA in blood serum. Additionally, Coolen et al. [65] used cationic peptide LAH4-L1 to encapsulate mRNA into LAH4-L1/mRNA polyplexes, then LAH4-L1/mRNA polyplexes were adsorbed onto poly(lactic acid) nanoparticles (PLA-NPs). PLA-NPs/LAH4-L1/mRNA nanocomplexes showed efficient gene transfection in DCs and activated innate and immune signaling responses [65]. Qiu et al. [27] introduced PEG modified cationic peptide KL4 (PEG_12_KL4) for pulmonary delivery of mRNA. PEG_12_KL4/mRNA complexes showed efficient transfection in human lung epithelial cells at a mass ratio of 10:1 (*w*/*w*). PEG_12_KL4/mRNA complexes were prepared into dry powder by spray drying (SD) and spray freeze drying (SFD) techniques. The intratracheal administration of PEG_12_KL4/mRNA complexes resulted in luciferase expression in the deep lung region of mice. The transfection efficiency of PEG_12_KL4/mRNA complexes was superior to Lipofectamine^TM^ 2000/mRNA complexes. Lou et al. [66] reported cationic peptide GALA-functionalized mRNA polyplexes (PPx-GALA) by copper-free click chemistry to enhance presentation of mRNA antigen by DCs. PPx-GALA formulation exhibited more efficient cellular uptake than lipofectamine-mRNA formulation. PPx-GALA containing mRNA encoding ovalbumin (OVA) showed enhanced T cell responses and DC maturation compared with free mRNA.

### Polymers

**Figure 4.**
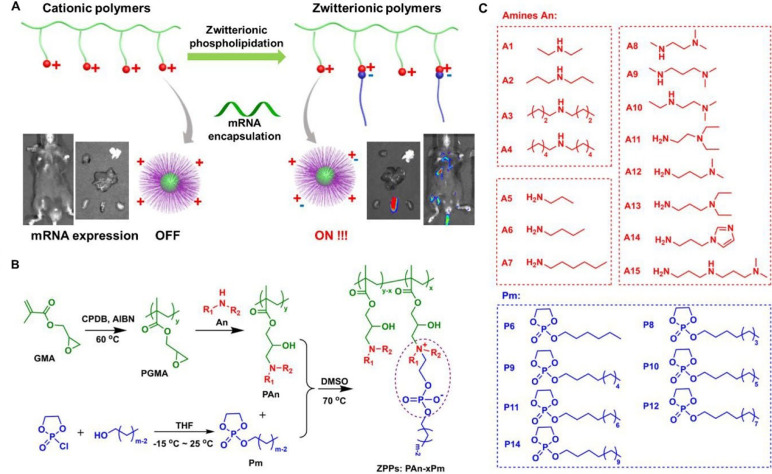
ZPPs used for efficient mRNA delivery into spleen and lymph nodes [70]. A. Phospholipidation transformed polycations to efficient zwitterionic mRNA carriers enabling protein expression in the spleen and lymph nodes; B. the synthetic route toward zwitterionic polymers PAn-xPm. “x” in “PAn-xPm” was defined as the number of Pm molecules functionalized on each polycation; C. fifteen An molecules and seven Pm molecules were used for combinatorial PAn-xPm synthesis. AIBN: azodiisobutyronitrile; An: amines; CPDB: 2-cyano-2-propyl benzodithioate; DMSO: dimethyl sulfoxide; GMA: glycidyl methacrylate; PGMA: poly(glycidyl methacrylate); Pm: alkylated dioxaphospholane oxide molecules; THF: tetrahydrofuran *Note*. Adapted from “Zwitterionic phospholipidation of cationic polymers facilitates systemic mRNA delivery to spleen and lymph nodes,” by Liu S, Wang X, Yu X, Cheng Q, Johnson LT, Chatterjee S, et al. J Am Chem Soc. 2021;143:21321–30 (https://pubs.acs.org/doi/pdf/10.1021/jacs.1c09822). Copyright © 2021 American Chemical Society.

Polymers with high molecular weight (usually to 10^4^–10^6^ dalton) could effectively load negatively charged nucleic acids by electrostatic interaction, so as to promote the nucleic acids delivery [3, 15–18]. In general, most polymeric carriers could improve the transfection efficiency and stability of mRNA. Zhao et al. [67] modified polyethyleneimine with stearic acid [polyethyleneimine-stearic acid (PSA)] and PSA could self-assemble to form polymeric micelles. PSA/mRNA nano-micelles could efficiently deliver mRNA and induce antigen-specific immune response. Blakney et al. [68] synthesized mannosylated polyethyleneimine by the host-guest interaction to deliver self-amplifying mRNA (sa-mRNA). Mannosylation of polyethyleneimine increased the percentage of transfected cells *ex vivo*. Additionally, mannosylated polyethyleneimine could promote the protein expression in the epithelial cells resident in human skin [68]. Vogel et al. [69] used medium-length polyethylenimine (PEI) to deliver synthetic mRNA and sa-mRNA expressing influenza virus hemagglutinin. They found that sa-mRNA was more effective than mRNA in protection against infection, and sa-mRNA combined in a trivalent formulation could protect against sequential H1N1 and H3N2 challenges. Liu et al. [70] synthesized zwitterionic phospholipidated polymers (ZPPs) to deliver mRNA to spleen and lymph nodes (Figure 4). This modular modification approach produced tunable zwitterionic species for serum resistance and introduction of alkyl chains simultaneously enhanced endosomal escape, which transformed deficient polymers into efficient zwitterionic mRNA carriers.

### Other types of carriers

As DCs could present antigen to T cells, DCs were utilized to deliver mRNA vaccine candidates. DCs could phagocytize pathogens and present the antigens to CD8^+^ and CD4^+^ T cells mediated by major histocompatibility complexes (MHC) class I and class II. Therefore, the *ex vivo* mRNA vaccine loading into DCs, and then reinfusion of transfected DCs into autologous recipient could induce an immune response. The *ex vivo* loading of mRNA by DCs was achieved by electroporation or lipid-derived carriers [71, 72]. This approach mainly exhibited cell-mediated immunity and was used for cancer immunotherapy [73]. Moreover, cationic nano emulsions were in oil in water emulsion-based delivery system. MF59, an oil in water emulsion adjuvant, was extensively studied for influenza vaccine development [35]. MF59 contained squalene droplets (4.3%) and surfactants like Tween 80 (0.5%) and Span85 (0.5%). To effectively encapsulate mRNA into cationic nano emulsions, DOTAP was the key component, which was used to complex with mRNA. Cationic nano emulsions not only accelerated the intracellular delivery of the mRNA, but also protect it against RNases degradation [74].

As the most widely used mRNA carriers, LNPs had high encapsulation efficiency and could exhibit efficient mRNA transfection *in vitro* and *in vivo*. In addition, LNPs had strong penetration into tissues and could achieve organ-specific targeting by regulating lipid components. Moreover, LNPs exhibited low cytotoxicity and immunogenicity. However, because LNPs contained a lot of components such as ionizable lipid (or cationic lipid), phospholipids, PEG-lipids, and cholesterol, the preparation process was complicated; microfluidic devices were usually required for preparation. Cationic peptides could be grafted with functional groups or modified with CPPs, which contributed to the cellular uptake and promoted mRNA transfection. However, cationic peptides needed to be prepared by solid-phase peptide synthesis. Additionally, a single cationic polypeptide could only be used for intracellular mRNA delivery; to achieve *in vivo* mRNA delivery, cationic lipids were usually modified with PEG segment. For polymers, the simple preparation process, abundant sources, and mass production had attracted widespread attention. However, compared with Lipos, transfection efficiency of polymers needed to be further improved.

## Routes of administration

Administration routes of mRNA therapeutics could greatly influence organ distribution and the therapeutic outcomes of carrier/mRNA complexes [75, 76]. The route of administration was usually determined by the properties of carriers and therapeutic indications. In general, i.v. administration of LNPs mainly accumulated in the liver after i.v. injection because of the discontinuous hepatic vasculature [77]; nanoscale diameters of LNPs [78] targeted the liver by adsorption of apolipoprotein E [79, 80], and LNPs could be metabolized by specified lipid metabolism pathways. Therefore, i.v. administration of LNPs mainly accumulated in the liver [81]. The i.v. administration of LNPs-mRNA could be used to express proteins that were missing in inherited metabolic and hematological diseases or produce antibodies that neutralized pathogens or acted on target cells [82–84]. The i.v. administration of LNPs-mRNA could accumulate in multiple lymph nodes throughout the body. Compared with local injection, the i.v. administration of mRNA vaccines was able to induce stronger antigen-specific cytotoxic T lymphocyte response [40, 85]. Topical administration methods had been used for mRNA therapeutics. Topical administration of carrier/mRNA complexes could accomplish local therapeutic effects. Local injection enables the supplementation of therapeutic proteins in specific organs including heart [86, 87], eyes [88, 89], and brain [90]. Additionally, the local injection could also evoke systemic responses, for example, i.d., i.m., and s.c. had been used for vaccination [20, 91], because the resident and recruited antigen-presenting cells (APCs) existed in skin and muscle, which could internalize and express mRNA-encoded antigens. Furthermore, the vascular and lymphatic vessels also helped APCs and mRNA vaccines migrate to drain lymph nodes and stimulate T cell immunity [20, 91]. Both i.d. and i.m. administrations of mRNA vaccines were able to induce robust immune response at a well-tolerated dose [92, 93]. Additionally, LNPs-mRNA complexes could be intratumorally injected into tumor tissues to boost a local pro-inflammatory environment, which resulted in immune cell activation and systemic anticancer responses [94, 95].

## Stability and storage of mRNA therapeutics

Storage of mRNA therapeutics needed to be considered before clinical translation, because the storage in an aqueous, freezing, or lyophilized manner and the category of cryoprotectants (sucrose, trehalose, or mannitol) affected the long-term stability of carrier/mRNA formulations [96]. The addition of sucrose or trehalose to mRNA therapeutics, stored in liquid nitrogen, could maintain delivery efficacy of mRNA for at least 3 months *in vivo* [96]. It was worth mentioning that the authorized COVID-19 mRNA vaccines were stored in freezing conditions using sucrose as a cryoprotectant [20]. mRNA-1273 was stored at –15°C to –20°C and injected after thawing, while BNT162b2 needed to be stored at –60°C to –80°C, and required thawing and dilution with saline before administration [20]. Although the cold-chain transportation-maintained mRNA vaccine activity, it also caused high transportation costs and delayed the vaccination process. Therefore, it was necessary to develop the technologies that did not need freezing or low-temperature storage for mRNA therapeutics.

## Safety of mRNA therapeutics

The safety profile of carrier/mRNA formulations was closely related to carriers and mRNA molecules. For LNPs, lipid components may activate host immune response by systemic or topical administration. For example, PEG-lipids tended to induce hypersensitivity reactions through stimulating the complement system [97]. Moreover, the production of anti-PEG antibodies would result in fast clearance of PEGylated nanoparticles during the blood circulation. To relieve safety concerns, some natural and synthetic polymers served as alternatives [97]. Additionally, cationic and ionizable lipids had been reported to arouse the secretion of pro-inflammatory cytokines and reactive oxygen species (ROS) [98–100]. Moreover, cytotoxicity of carriers also needed attention, and it was reported that the *in vivo* application of LNPs might cause liver and lung injuries in rodents. Furthermore, the immunogenicity of *in vitro* transcribed mRNA was also a safety concern, because immune response of *in vitro* transcribed mRNA may suppress antigen expression and attenuate vaccine efficacy. To minimize the immunogenicity of mRNA, chemical modification of specific *in vitro* transcribed mRNA nucleotides and chromatographic purification were carried out [101, 102].

## mRNA drugs for disease treatment

### Infectious diseases

Influenza was usually caused by viruses; mRNA vaccines could stimulate both cellular and humoral immunity, protecting the human body from the infection of influenza (Table 1). In 2019, COVID-19 broke out worldwide, causing huge economic losses and health threats. To effectively prevent COVID-19, Pfizer/BioNTech and Moderna developed mRNA vaccines NT162b2 and mRNA-1273, respectively, which obtained urgent authorization from FDA. Both NT162b2 and mRNA-1273 were associated with some allergic symptoms including pain, redness, and fever, and BNT162b2 had lower adverse reactions than mRNA-1273 [103].

**Table 1.** mRNA drugs used for prevention and treatment of diseases

**Disease type**	**Name**	**Administration route**
Infectious diseases		
COVID-19	NT162b2	i.m.
	mRNA-1273	
Rabies	RVG-SAM	i.m.
Cancer		
A20-OVA lymphoma	OVA mRNA	i.v.
B16F10-OVA melanoma	OVA mRNA	i.v.
Hepa1-6 orthotopic HCC tumor	BisCCL2/5i mRNA	i.v.
Cardiovascular diseases		
Familial hypercholesterolaemia and atherosclerotic cardiovascular diseases	mRNA encoding Cas9	i.v.
Type 2 diabetes	VEGF-A mRNA	i.d.
Genetic disorder		
Monogenic retinal degenerative disorders of retinal pigmented epithelium	EGFP mRNA	Subretinal injection
Methylmalonic acidemia	mRNA encoding human wild-type MCMUT	i.v.
Fabry disease	α-Gal A mRNA	i.v.

CCL2: C-C motif chemokine ligand 2; EGFP: enhanced green fluorescence protein; HCC: hepatocellular carcinoma; MCMUT: methylmalonyl-coenzyme A (CoA)-mutase; RVG-SAM: self-amplifying RNA encoding the rabies virus glycoprotein; VEGF-A: vascular endothelial growth factor A; α-Gal A: alpha-galactosidase A

Moreover, mRNA vaccines have been used for other kinds of pathogens. For example, Castanha et al. [104] indicated mRNA vaccines were efficient against rabies in rodents. During the observation period, mRNA vaccine could induce sufficient specific CD4^+^ T cells and the neutralizing antibody titers were stable. Lou et al. [105] used LNPs loading mRNA vaccine to mediate rabies virus glycoprotein expression and LNPs loading mRNA vaccine showed a good protection effect.

### Cancer

Cancer, as a malignant disease, was a great threat to human life and health [3, 106–108]. In view of the unique advantages of mRNA therapeutics, researchers began to use mRNA technology to prevent and treat cancer. For example, to enhance the *in vivo* delivery efficiency of mRNA encoding antigen, Haabeth et al. [8] designed charge-altering releasable transporters to mediate mRNA therapeutics to APCs. They found that carrier/mRNA vaccine could induce a strong antigen-specific immune response in peripheral blood mononuclear cells. Moreover, this mRNA vaccine could efficiently target APCs in secondary lymphoid organs after intravenous injection and induced an anti-tumor immune response in the established tumors. For another example, to accelerate the intracellular delivery of mRNA for antigen translation and activate an appropriate immune response, Miao et al. [109] developed a combinatorial library of ionizable lipid-materials. The top candidate formulation could induce a robust antitumor immune response and significantly inhibit tumor growth and prolong the survival in tumor models [109]. Moreover, liver malignancy was resistant to immune checkpoint blockage, Wang et al. [110] found that *CCL2* and *CCL5* were the main chemokines attracting tumor-associated macrophages infiltration and resulted in the resistance to immune checkpoint inhibition. To relieve the immunosuppression, mRNA encoding single-domain antibody that specifically neutralized *CCL2* and *CCL5* encapsulated in clinically approved LNPs, which resulted in efficient antibody expression in the diseased organ. The mRNA therapy combining with programmed cell death protein 1 (*PD-1*) ligand inhibitor considerably improved the survival in liver cancer-bearing mice model and could reduce liver metastasis in colorectal and pancreatic cancers. Furthermore, to achieve the goal of the tumor prevention and treatment simultaneously, Islam et al. [111] constructed adjuvant plus mRNA vaccine nanoparticles, which was coated with lipid-PEG. This adjuvant plus mRNA vaccine could activate adaptive immune response by improving the proportion of OVA-specific CD8^+^ T cells. This strategy showed an excellent anti-tumor effect in OVA-expressing lymphoma and prostate cancer-bearing syngeneic allograft mouse models and significantly prevented tumor growth once vaccine was injected before tumor implantation.

### Cardiovascular disease

Cardiovascular disease was another major disease threatening people’s life and health, especially for middle-aged and elderly patients, with a very high mortality. Despite remarkable advances obtained in prevention and treatment, cardiovascular disease was one of the leading causes of death worldwide [112, 113]. One of the targets of cardiovascular disease was the high content of proprotein convertase subtilisin/kexin type 9 (PCSK9), leading to improved cholesterol in the body and inducing familial hypercholesterolaemia and atherosclerotic cardiovascular diseases [114]. Cheng et al. [12] used LNPs to co-deliver mRNA encoding *Cas9* and sgRNA targeting *PCSK9 in vivo*. They found preferred LNPs remarkably induced the loss in PCSK9 locus and resulted in considerable downregulation of PCSK9 protein levels in liver [12]. In addition, except for inhibiting endogenous mRNA of PCSK9, a cardiovascular-related mRNA therapeutic namely, mRNA encoding *VEGF-A*, had entered the clinical trials [115]. The intradermal administration of mRNA encoding *VEGF-A* considerably increased the expression of VEGF-A protein and improved skin blood flow in male patients with type 2 diabetes.

### Genetic disorder

Exogenous mRNA therapeutic was emerging as a novel medicine of broad applicability in a variety of diseases including monogenic disorders [116]. This was because the delivered mRNA was able to encode a variety of functionally therapeutic proteins. For example, Patel et al. [88] developed a library of LNPs to mediate mRNA delivery into the back of the eye. The preferred LNPs showed efficient gene transfection in the retina. Moreover, LNPs loaded mRNA could effectively relieve monogenic retinal degenerative disorders of retinal pigmented epithelium. For another example, Sabnis et al. [51] used LNPs loaded mRNA encoding human wild-type MCMUT for treating methylmalonic acidemia. Such LNPs loaded mRNA encoding MCMUT could dramatically improve the survival and down-regulate the disease biomarkers in murine models of methylmalonic acidemia. Fabry disease was an X-linked lysosomal storage genetic disorder due to the mutation of galactosidase A gene (*GLA*), encoding the lysosomal enzyme α-Gal A. Zhu et al. [117] encapsulated α-Gal A mRNA with LNPs and injected it intravenously. A duration effect was observed after a single dose of mRNA therapeutic in Fabry mice model as confirmed by the glycosphingolipids reductions in disease site.

## Conclusions

Due to the COVID-19 pandemic, severe acute respiratory syndrome coronavirus 2 (SARS-CoV-2) mRNA vaccines have come into people’s view, such as the emergency approval of Pfizer/BioNTech and Moderna mRNA vaccines. This review described the types of mRNA carriers, routes of mRNA administration, and storage of mRNA therapeutics, and the type of diseases for which mRNA drugs were appliable was discussed in detail.

Advances in mRNA technology and gene delivery have led to the rapid development of mRNA vaccines, which also proves the potential of mRNA drugs in clinical application. At present, researchers have developed and optimized a variety of LNPs and other types of mRNA carriers, providing beneficial guidance for mRNA drug-based prevention and treatment. According to the experience and lessons of clinical data, the mRNA formulations needed to be optimized. Firstly, the *in vitro* and *in vivo* delivery efficiency of mRNA needs to be further improved. For LNPs carriers, modulating the head groups and hydrophobic tails can contribute to the cellular uptake of mRNA, and finally promotes the efficacy of mRNA therapeutics. Additionally, hybrid nanocarriers can also be constructed by combining lipid nanocarriers and cationic polymers or cationic peptides. The hybrid nanocarriers can access to the advantages of individual carriers for improving mRNA delivery efficacy.

Moreover, regulating lipid structure can realize organ-specific delivery of LNPs. For example, modulation of lipid alkyl length led to the selective accumulation of LNPs in liver or spleen. Zwitterionic phospholipidation of cationic polymers can selectively accumulate in the spleen after systematic administration. Moreover, neurotransmitters, as endogenous chemicals, can cross the blood-brain barrier and participate in neurotransmission. In addition, neurotransmitter-derived lipids can be used for mRNA transmission to the brain after intravenous injection. Neurotransmitter-derived lipids can be used for mRNA delivery to the brain after intravenous administration.

At present, the clinical mRNA therapeutic agents were mainly LNPs, and researchers have developed a variety of mRNA administration routes. In general, mRNA therapeutic agents have poor stability at room temperature and need to be stored at low temperatures, which also leads to additional storage and transportation costs. Additionally, safety of mRNA therapeutic agents also arouses clinical concern. Biodegradability and versatility needed to be considered during development of mRNA therapeutic agents. High efficiency, satisfactory safety, stable storage at room temperature, biodegradability, and versatility will be the directions of the development of mRNA therapeutics in the future.

## Abbreviations

APCs: antigen-presenting cells
CCL2: C-C motif chemokine ligand 2
COVID-19: coronavirus disease 2019
CPPs: cell-penetrating peptides
DCs: dendritic cells
DLin-MC3-DMA: dilinoleylmethyl-4-dimethylaminobutyrate
DOPE: 1,2-dioleoylphosphatidylethanolamine
DOTAP: 1,2-dioleoyl-3-trimethylammonium-propane
DOTMA: *N*-[1-(2,3-dioleyloxy)propyl]-*N*,*N*,*N*-trimethylammonium chloride
DSPC: 1,2-distearoyl-*sn*-glycero-3-phosphocholine
FDA: Food and Drug Administration
Lipo: liposome
LNPs: lipid nanoparticles
LPR: liposome-protamine-RNA
MCMUT: methylmalonyl-coenzyme A-mutase
mRNA: messenger RNA
OVA: ovalbumin
PCSK9: proprotein convertase subtilisin/kexin type 9
PEG: polyethylene glycol
PEG_12_KL4: polyethylene glycol modified cationic peptide KL4
PEG-lipids: polyethylene glycol-functionalized lipids
pK_a_: acid dissociation constant
PPx-GALA: GALA-functionalized messenger RNA polyplexes
PSA: polyethyleneimine-stearic acid
sa-mRNA: self-amplifying messenger RNA
VEGF-A: vascular endothelial growth factor A
ZPPs: zwitterionic phospholipidated polymers
α-Gal A: alpha-galactosidase A

## Declarations

### Copyright

© The Author(s) 2022.
